# Dysfunction of DMT1 and miR-135b in the gut-testis axis in high-fat diet male mice

**DOI:** 10.1186/s12263-024-00737-6

**Published:** 2024-01-19

**Authors:** Yanru Zhang, Ruike Ding, Yulin Zhang, Jia Qi, Wenbin Cao, Lijun Deng, Lin Zhou, Yun Ye, Ying Xue, Enqi Liu

**Affiliations:** 1 Laboratory Animal Center, Xi’an Jiaotong University Health Science Centre, Xi’an, 710061 China; 2 Spring Biological Technology Development Co., Ltd, Fangchenggang, Guangxi, 538000 China; 3 Central Laboratory, The First Affiliated Hospital of Xi’an Medical University, Xi’an, 710000 China; 4 Key Laboratory of Environment and Genes Related to Diseases, Ministry of Education of China, Xi’an, 710049 China

**Keywords:** Obesity, Iron deficiency, DMT1, Epigenetic, MicroRNAs

## Abstract

**Background:**

Obese patients have been found to be susceptible to iron deficiency, and malabsorption of dietary iron is the cause of obesity-related iron deficiency (ORID). Divalent metal transporter 1 (DMT1) and ferroportin (FPN), are two transmembrane transporter proteins expressed in the duodenum that are closely associated with iron absorption. However, there have been few studies on the association between these two proteins and the increased susceptibility to iron deficiency in obese patients. Chronic inflammation is also thought to be a cause of obesity-related iron deficiency, and both conditions can have an impact on spermatogenesis and impair male reproductive function. Based on previous studies, transgenerational epigenetic inheritance through gametes was observed in obesity.

**Results:**

Our results  showed that obese mice had decreased blood iron levels (*p* < 0.01), lower protein and mRNA expression for duodenal DMT1 (*p* < 0.05), but no statistically significant variation in mRNA expression for duodenal FPN (*p* > 0.05); there was an increase in sperm miR-135b expression (*p* < 0.05). Bioinformatics revealed ninety overlapping genes and further analysis showed that they were primarily responsible for epithelial cilium movement, fatty acid beta-oxidation, protein dephosphorylation, fertilization, and glutamine transport, which are closely related to spermatogenesis, sperm development, and sperm viability in mice.

**Conclusions:**

In obese mice, we observed downregulation of DMT1 in the duodenum and upregulation of miR-135b in the spermatozoa.

## Background

Obesity has become a global epidemic that endangers human health and increases the medical burden on society. Body mass index (BMI) is used to estimate a person’s weight category (normal/overweight/obese), and in 2016, 39% of individuals above the age of 17 were considered to be overweight (BMI ≥ 25), while 13% were obese (BMI ≥ 30) [1]. Current data suggest that by 2038, over one-third of the global population will be obese [2]. According to a 1961 study by Wenzel et al. [3], obese individuals had lower serum iron concentrations than non-obese persons and this has subsequently been supported by the findings of other researchers [4]. There has been an increase in studies showing that obesity affects micronutrient absorption, especially iron, which can lead to iron deficiency or even iron deficiency anemia [5–7]. Dietary absorption of stable isotope tracer iron was significantly lower in obese children than in non-obese control children [8], suggesting that dietary iron malabsorption could be an important cause of obesity-related iron deficiency.

Iron is an essential trace element for the human body and an essential biological element for almost all organisms, as it is involved in such biological processes as transport and storage of oxygen and electron transfer. Iron deficiency can lead to immune system disorders, and anemia, and even affect growth and motor development in children [9]. The duodenum is one of the main sites of iron absorption and is critical for the regulation of iron homeostasis [10]. Iron in food is mainly in the form of Fe^3+^, which is reduced to Fe^2+^ by duodenal cytochrome b (Dcytb) located on the duodenal parietal membrane. DMT1, a transmembrane transporter protein on the duodenal parietal membrane, then transports Fe^2+^ into the cell [11–14]. Iron entering small intestinal cells is released after passing through another transmembrane transporter protein, FPN, which is the only known channel protein for the extracellular transport of non-heme iron and plays an important role in development and intestinal iron absorption in mice [15].

Obesity is characterized by an excessive accumulation of fatty adipose tissue. Immune cells such as macrophages and lymphocytes in adipose tissue can be activated and release inflammatory factors like TNF-α, leading to chronic inflammation [16]. Chronic inflammation is also associated with anemia and is one of the reasons why obese patients are prone to iron deficiency [17]. Hepcidin is a small peptide secreted by the liver that inhibits intestinal iron absorption and helps to regulate iron homeostasis. Chronic inflammation produces the pro-inflammatory cytokine, interleukin-6 (IL-6), which induces the production of hepatic hepcidin. Therefore, some investigators have suggested that inflammation-induced upregulation of hepcidin expression may be a potential modulator between obesity and iron deficiency [18–22]. Pro-inflammatory cytokines may also affect erythropoietin levels and increase iron retention by reticuloendothelial cells, leading to anemia in some obese individuals [23]. ORID and abnormal iron absorption have many adverse effects on the male reproductive system. Iron deficiency can impair sperm development and increase oxidative damage to sperm [24] as well as reduce ejaculate volume, sperm count, and sperm viability [25, 26]. Studies have shown that although serum iron is lower in obese people, iron levels in seminal plasma and spermatozoa are high [27]; however, higher iron levels in seminal plasma are negatively associated with male fertility, and the adverse effects may be mediated by oxidative stress [28].

RNA is one of the epigenetic markers of germ cells, and phenotypic changes resulting from exposure of the father to certain environmental factors can be inherited by the offspring through sperm RNAs. For example, western diet-induced phenotypic inheritance can be mediated through sperm RNAs [29]. Small non-coding RNAs (sncRNAs) can transmit information about environmental factors and are carriers of environmental influences to the genome [30]. These sncRNAs are abundant in sperm and play an important role in cross-generational inheritance [31]. MicroRNAs (miRNAs) are a group of sncRNAs 20–23 nucleotides in length encoded by the genome, which directs the RNA-induced silencing complex (RISC) to degrade mRNAs or block their translation by base-pairing with target gene mRNAs. There are many miRNAs such as miR-135b that have been shown to be closely associated with inflammation, and interleukin 1 can upregulate miR-135b expression to promote inflammation [32].

To investigate whether the ORID is associated with duodenal DMT1 and FPN expression, we used a high-fat diet-induced obese mouse model. The serum iron levels of the obese mice were determined and the expression levels of duodenal DMT1 and FPN were assessed by immunohistochemical methods and quantitative real-time polymerase chain reaction (qRT-PCR). The expression of sperm miR-135b in obese mice was also measured by qRT-PCR, and the data were subjected to bioinformatics analysis. Consistent with human data, we confirmed that serum iron levels were also lower in obese mice and that lower DMT1 mRNA and protein levels could inhibit duodenal iron absorption. Due to its positive correlation with inflammation, the high expression of miR-135b in the sperm of obese mice suggested that the sperm were indeed affected by inflammation. Further bioinformatics analysis revealed that miR-135b affected fertility in obese mice, including spermatogenesis, sperm development, and fertilization.

## Materials and methods

### Animal experiments and a mouse model of obesity

The animal protocols and care were in accordance with the guidelines of Xi'an Jiaotong University’s Animal Experimental Ethics Committee. The animals used in the experiments were provided by the Xi’an Jiaotong University Health Science Center. Twenty male-specific pathogen-free (SPF) ICR mice (5 weeks old) were used in this study. After 1 week of acclimatization, the mice were randomly separated into two groups of ten each. The control group was fed the standard diet (SD) that provided 10% fat energy (DIO12450B, Beijing Keao Xieli Feed Co., Ltd). The model group received a high-fat diet (HFD) containing a 60% fat-energy ratio chow (DIO12492, Beijing Keao Xieli Feed Co., Ltd.). The mice were fed for nine weeks and their body weight was measured once a week. The temperature and humidity were controlled, and the mice were kept on a 12-h light/12-h dark cycle each day.

### Collection of blood and other samples

After feeding, mice were fasted for 6 h, anesthetized using tribromoethanol (M2920, Jiangsu Yihe Scientific Instrument Co., China), and then blood was drawn. Subsequently, the mice were euthanized by cervical dislocation, after which their duodenum, testes, and bilateral epididymal tails were immediately removed. After standing for 30 min, the blood was centrifuged at 1800 × *g*, the supernatants were collected into fresh tubes, centrifuged a second time at 1800 × *g* for 5 min, and the supernatants were collected again after centrifugation to obtain the serum. For sperm collection, the epididymal tails were clipped and placed in 1.5 ml of PBS (130 mM NaCl, 10 mM Na_2_PO4, 1.7 mM KH_2_PO4, 2 mM KCl, pH 7.4) and kept at 37 °C for 15 min to allow sperm release. The suspension was filtered through a 40-μm cell filter to remove tissue pieces, and the filtrate was mixed with 10 volumes of somatic cell lysis solution (0.1% SDS, 0.5% Triton X-100, DEPC H_2_O), and kept on ice for 40 min after mixing for 5 min. A visible light microscope was used to confirm that the somatic cells were all lysed. The suspension was centrifuged at 10,000 × *g* for 8 min at 4 °C, and the sperm pellet was washed twice with PBS and again centrifuged under the same conditions. The supernatant was removed and TRIzol reagent was added for RNA extraction. After duodenal samples were collected, one part was placed into nuclease-free tubes with TRIzol reagent for RNA extraction, and the other part and the testes were placed in 4% paraformaldehyde and fixed at room temperature for 24 h.

### Serum iron assay

Serum iron content was determined using a serum iron assay kit (A039-1–1, Nanjing Jiancheng Bioengineering Institute, China), following kit instructions. The absorbance was detected at 520 nm with a microplate spectrophotometer.

### Hematoxylin and Eosin (H&E) and immunohistochemistry staining

After fixation, the duodenum and the testes samples were rinsed with running water for 30 min, then dehydrated in graded ethanol and cleared. After being embedded in paraffin, samples were sliced into 5-μm sections, dewaxed with xylene for 10 min, and rinsed in 100%, 95%, and 90% ethanol for 3 min.

For HE staining, the sections were dehydrated after staining with HE reagent and then sealed with a neutral fixative. For immunohistochemistry staining, sodium citrate antigen epitope retrieval solution (MVS-0101, Fuzhou Maixin Biotech, China) was used for antigen retrieval by heating in a microwave. The sections were next blocked with 10% goat serum (AR0009, Boster Biological Technology, China) at 37 °C for 30 min. This treatment reduces the non-specific binding of the subsequent primary and secondary antibodies to the samples and lowers the background. Primary antibody immunostaining solution (P0262, Shanghai Biyuntian Biotechnology, China) was used to dilute the DMT1 antibody (A10231, ABclonal Technology, China) at a ratio of 1:200, and sections were transferred to wet boxes for overnight incubation with primary antibody at 4 °C. Sections were rinsed three times with PBS. Immunostaining secondary Ab diluent (P0265, Shanghai Biyuntian Biotechnology, China) was used to dilute HRP-conjugated goat anti-rabbit IgG H&L at a ratio of 1:200. After adding secondary Ab, the sections were transferred to the wet box for 1 h at 37 ℃, then rinsed with PBS. Sections were stained with DAB for 5 s and then with hematoxylin for the nucleus. After rinsing, the sections were dehydrated, covered with neutral fixative, digitally imaged under a visible light microscope, and analyzed.

### RNA extraction and qRT-PCR

RNA was extracted from duodenal and sperm samples using the TRIzol method, and RNA concentrations and A260/A280 were measured using a UV spectrophotometer. Complementary DNA (cDNA) was synthesized from 1 μg of total duodenal RNA using the Evo M-MLV RT mix kit with gDNA-Clean for qPCR (AG11728, Accurate Biology, China). The miRNA 1st strand cDNA synthesis kit (stem-loop) (AG11743, Accurate Biology, China) was employed to synthesize cDNA from 200 ng of gross sperm RNA from sperm samples. SYBR® Green Premix Pro Taq HS qPCR kit (AG11701, Accurate Biology, China) was used to measure mRNA expression levels in a 20-μL total reaction system. Glyceraldehyde-3-phosphate dehydrogenase (GAPDH) was used as the internal normalization control for duodenal RNA and U6 as the internal normalization control for sperm RNA. Table 1 lists the qRT-PCR primer sequences, which were synthesized by the Tsingke Biotechnology Co. (https://tsingke.com.cn/).

**Table 1:** Primers used for gene expression assay by RT-qPCR

Primer name	Primer sequence
GAPDH F	AGGTCGGTGTGAACGGATTTG
GAPDH R	TGTAGACCATGTAGTTGAGGTCA
DMT1 F	TCTATGTGGTGGCTGCAGTG
DMT1 R	GCTGGTATCTTCGCTCAGCA
FPN F	GGCACTTTGCAGTGTCTGTG
FPN R	GTCACCAATGATGGCTCCCA
Stem-loop F	ATCCAGTGCGTGTCGTG
miR-135b RT	GTCGTATCCAGTGCGTGTCGTGGAGTCGGCAATTGCACTGGATACGACTCACATA
miR-135b R	TGCTTATGGCTTTTCATTCC
U6 RT	GTCGTATCGACTGCAGGGTCCGAGGTATTCGCAGTCGATACGACAAAAATAT
U6 F	AGCACATATACTAAAATTGGAACGAT
U6 R	ACTGCAGGGTCCGAGGTATT

### MiRNA target gene prediction and functional annotation

The miRWalk online tool (http://mirwalk.umm.uni-heidelberg.de/) was used to predict the target genes of miR-135b. Relevant keywords were entered in the National Center of Biotechnology Information (NCBI) Gene Expression Omnibus (GEO; http://www.ncbi.nlm.nih.gov/geo/) database, and the GSE133915 dataset was identified and downloaded. The mRNA expression database included 16 samples, eight sperm samples for the HFD group and eight sperm samples for the SD group. The Xiantao Academic online tool (https://www.xiantaozi.com/) was used for the DESeq2 analysis. The DESeq2 package was used to perform differential expression analysis on the original counts matrix, following the standard procedure, and the variance stabilizing transformations (VST) method provided by the DESeq2 package was used to normalize the original counts matrix. The screening conditions were |log2 (fold-change) |> 0.58 and *p* < 0.05, and 2149 differentially expressed genes (DEGs) were screened. The Venn diagram online mapping tool (http://bioinformatics.psb.ugent.be/webtools/Venn/) was used to indicate the overlaps of DEGs and predicted target genes. The database for annotation, visualization and integrated discovery (DAVID) (https://david.ncifcrf.gov/) was used for gene ontology (GO) and the Kyoto Encyclopedia of Genes and Genomes (KEGG) for pathway analysis of overlapping genes. Mouse genome informatics (MGI) (https://www.informatics.jax.org/) was used to search all genes related to the male sterile phenotype in mice and the gene ontology (GO) knowledgebase (http://geneontology.org/) was used to search for genes associated with fertilization, sperm development and spermatogenesis in mice.

### Statistical analysis

The image intensity of the duodenal DMT1 staining results was determined using Image-Pro Plus 6.0, 10 randomly chosen, 200 × fields per section were evaluated for each mouse. Data are mean ± standard error of the mean (SEM). SPSS was employed to analyze serum iron levels, DMT1, and FPN expression in the duodenum, and data were compared using Student’s *t* test. A value of *p* < 0.05 was regarded as significantly different in statistical terms.

## Results

### Abnormal testes morphology in obese mice

To establish the HFD-induced obesity model in mice, we divided the mice into two groups, SD and HFD, with feeding of 10% and 60% fat energy ratio, respectively. Initially, the mean body weights of mice were 23.8 g ± 0.95 in the HFD and 23.2 g ± 1.32 in the SD group. By the end of 9 weeks, the mean body weights of the mice in the HFD and SD groups were 42.38 g ± 2.18 and 32.08 g ± 1.38, respectively, and the body weights of the HFD mice exceeded the mean body weights of the SD mice by 20%.

**Fig. 1:**
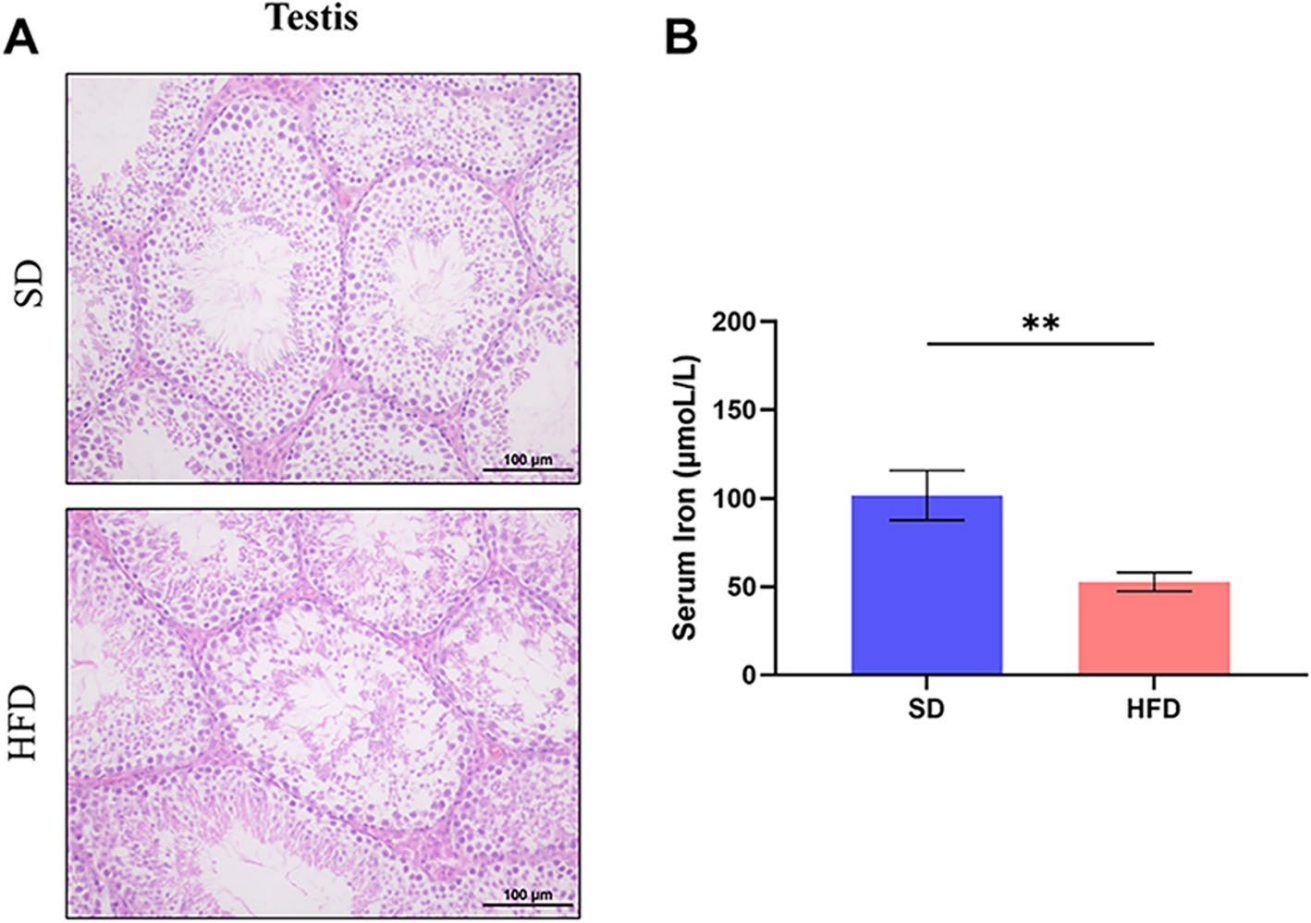
Effects of HFD on testicular morphology and serum iron levels. **A** Morphological analysis of testis from SD and HFD groups. **B** Serum iron levels were significantly decreased in HFD-fed obese mice compared to SD-fed control mice. Data are expressed as mean ± SEM; **p < 0.01

In our study, HE staining was performed on testicular sections from both obese mice and the control group to evaluate the potential impact of obesity on male reproductive function (Fig. 1A). In the SD control group, seminiferous tubules exhibited clear identification of spermatogonia, spermatocytes at various stages, and spermatozoa. In contrast, the HFD group displayed sparser cellular arrangement within the seminiferous tubules, reduced number of spermatogonia, thinning of the germinal epithelium, and a notable absence of spermatozoa in most tubules. In conclusion, the HE-stained testicular sections from the HFD group demonstrated several morphological abnormalities compared to the SD control group, suggesting that obesity adversely affects male reproductive function in mice.

### Serum iron

The serum iron levels in both groups of mice were measured, and the results showed that the serum iron levels were significantly lower in the obese mice compared with the SD control (Fig. 1B).

### Expression levels of duodenal DMT1 and FPN

**Fig. 2:**
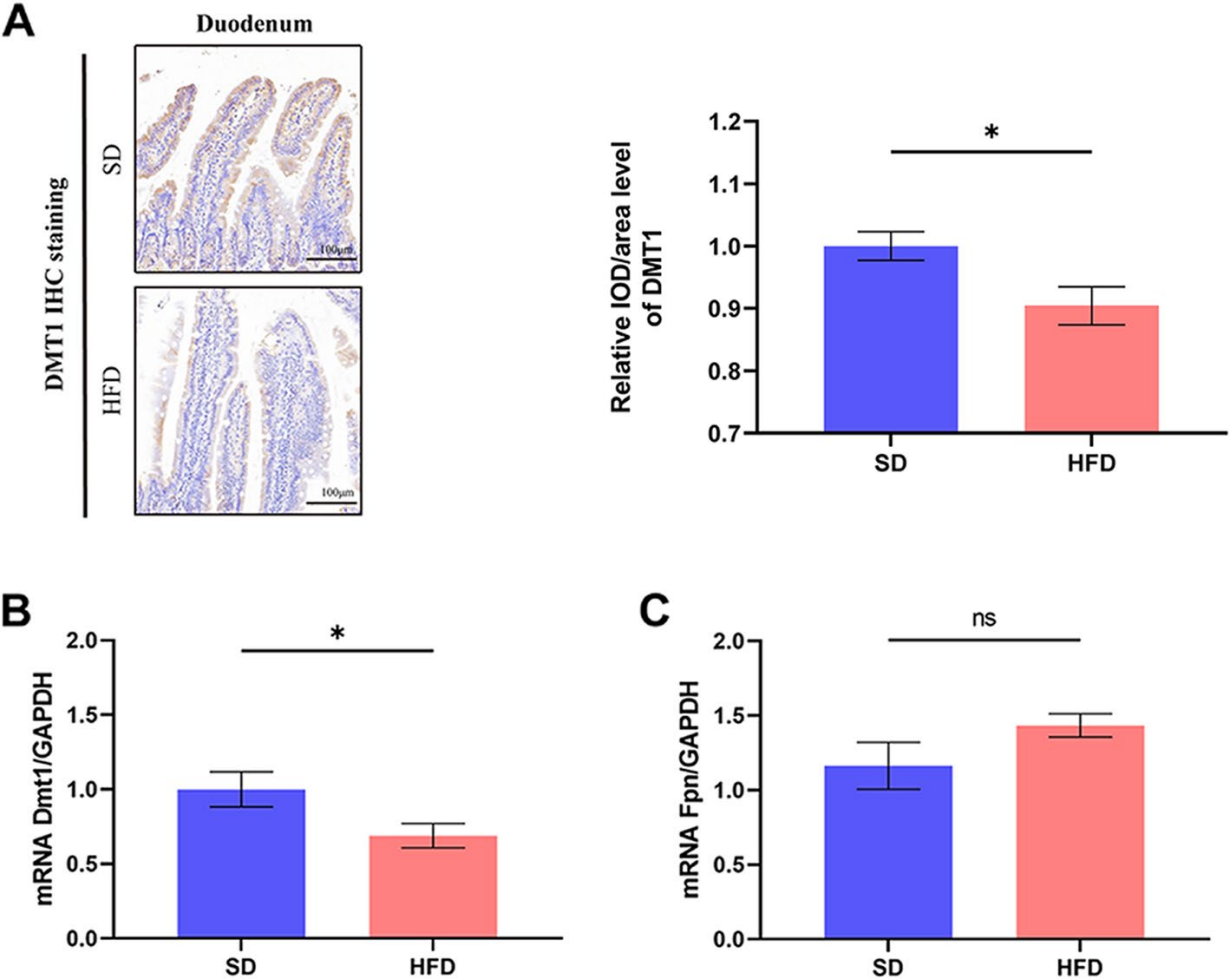
HFD-induced obesity reduces the protein and mRNA expression of duodenal DMT1. **A** Immunohistochemical staining of duodenal DMT1 in SD and HFD mice. The mRNA expression levels of **B** DMT1 and **C** FPN in SD and HFD mice. Data are expressed as SEM ± mean; *p < 0.05; *ns* indicates *p* > 0.05

To evaluate the impact of obesity on duodenal iron absorption, we determined the expression levels of duodenal DMT1 in the two groups of mice by immunohistochemistry and qRT-PCR. The DMT1 protein and mRNA levels were significantly decreased in the duodenum of HFD-induced obese mice compared to normal weight controls (Fig. 2A, B). The expression levels of FPN in the duodenum were also compared by qRT-PCR, which showed no difference in FPN mRNA expression in the duodenum (Fig. 2C).

### Expression of miR-135b in sperm and functional annotation of DEGs

**Fig. 3:**
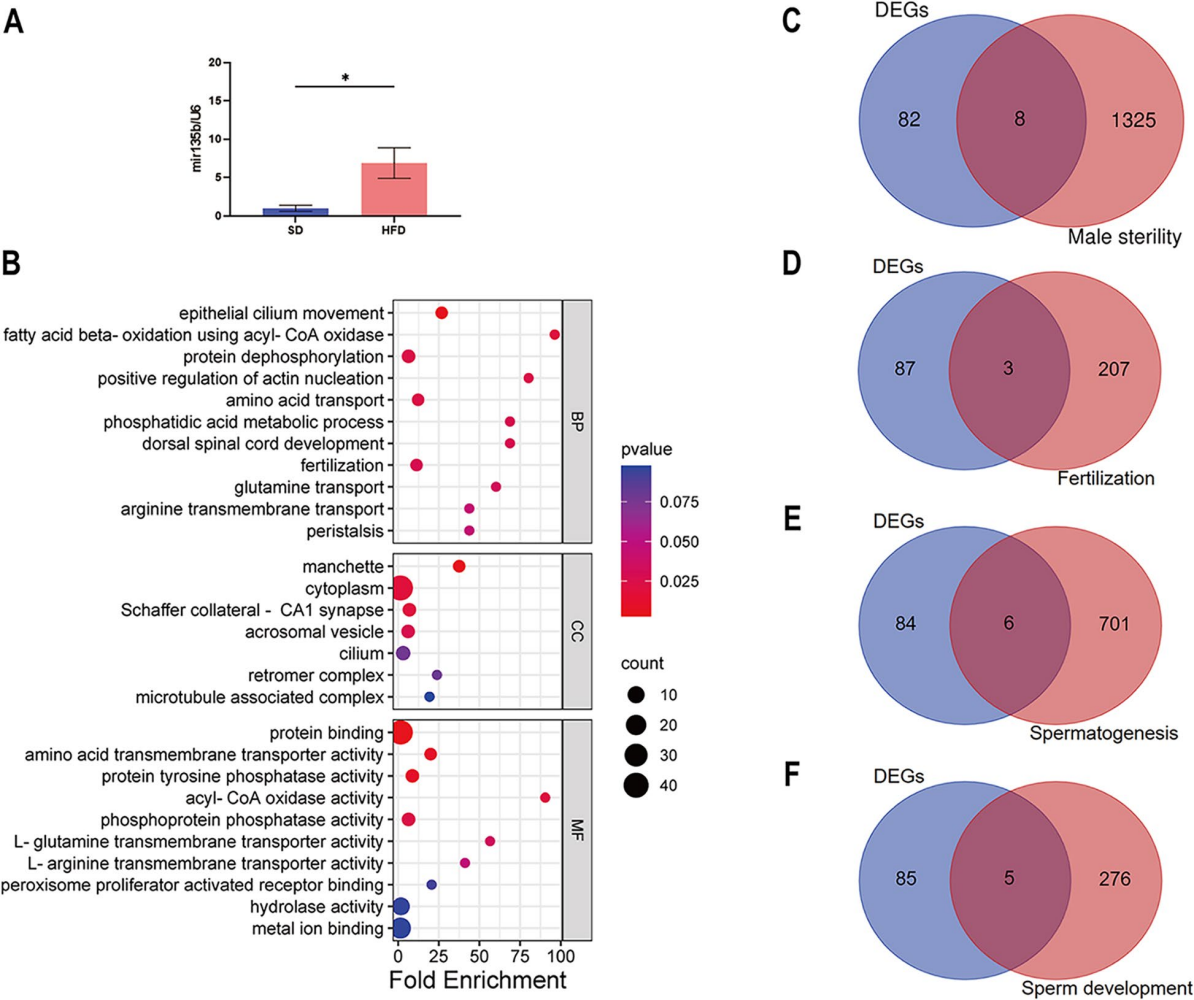
Expression of miR-135b in sperm and functional annotation of DEGs. **A** Expression of miR-135b from sperm of SD vs HFD mice; *p < 0.05. **B** Bubble diagram of GO for 90 DEGs. Venn diagrams of 90 DEGs and genes related to **C** male sterility phenotype, **D** fertilization, **E** spermatogenesis, and **F** spermatozoa development

The qRT-PCR method was performed to evaluate miR-135b expression in the spermatozoa of obese mice compared to normal-weight mice. The results (Fig. 3A) showed that the expression level of miR-135b was significantly increased in the spermatozoa of mice on the HFD compared with those fed the SD.

GSE133915 is the set of transcriptome data comparing the spermatozoa of mice in the SD group and HFD group by RNA sequencing. The Xiantao Academic online tool was used to perform DESeq2 analysis on the transcriptome data with a screening condition of |log2 (fold-change) |> 0.58 and *p* < 0.05. A total of 2149 DEGs were screened, of which 1924 genes were upregulated and 225 genes were downregulated. Target genes of miR-135b were predicted using the miRWalk platform. Overlapping genes of target genes and DEGs were shown as Venn diagrams using an online mapping tool, and a total of 90 genes were obtained, of which 79 were upregulated and 11 were downregulated. GO analysis (Fig. 3B) was used to determine the biological process (BP), molecular function (MF), and cellular component (CC) classifications of these 90 genes, and *p* < 0.05 was considered significantly enriched. The top 12 BPs were enriched in phosphatidic acid biosynthesis, epithelial cilium movement, fatty acid beta-oxidation using acyl-CoA oxidase, protein dephosphorylation, positive regulation of actin nucleation, amino acid transport, phosphatidic acid metabolism, dorsal spinal cord development, fertilization, glutamine transport, arginine transmembrane transport, and peristalsis. The CCs were enriched in spermatid manchettes, cytoplasm, Schaffer collateral-CA1 synapses, acrosomal vesicles, cilia, retromer complexes, and microtubule-associated complexes. The MFs were enriched in protein binding, amino acid transmembrane transporter activity, protein tyrosine phosphatase activity, acyl-CoA oxidase activity, phosphoprotein phosphatase activity, L-glutamine transmembrane transporter activity, L-arginine transmembrane transporter activity, peroxisome proliferator-activated receptor binding, hydrolase activity, and metal ion binding. Within these three elements, ninety DEGs were involved in fertilization and strongly associated with spermatogenesis, sperm development, and sperm viability. KEGG enrichment had no results.

The MGI database was used to search for genes related to the mouse male sterility phenotype, and the GO knowledge base was used to search for genes related to fertilization, sperm development, and spermatogenesis in mice. Overlaps among these genes and the 90 DEGs were obtained and depicted as Venn diagrams using an online mapping tool. There were 1334 genes related to male sterility phenotype in mice, and 8 genes overlapped with the 90 DEGs (Fig. 3C), namely *Spag17*, *Tdrd12*, *Stk36*, *Nxf2*, *Bard1*, *Lpin1*, *Spef2* and *Jag2*. There were 210 genes related to mouse fertilization and 3 genes overlapping with the 90 DEGs (Fig. 3D), namely *Tdrd12*, *Spesp1*, and *Fam170b*. There were 707 genes related to mouse spermatogenesis and 6 genes overlapping with the 90 DEGs (Fig. 3E), namely *Ica1l*, *Spag17*, *Lztfl1*, *Tdrd12*, *Spef2*, and *Jag2*. There were 281 genes associated with mouse spermatogenesis, and we found 5 genes overlapping with the 90 DEGs (Fig. 3F), namely *Ica1l*, *Spesp1*, *Adam30*, *Spag17*, and *Spef2*.

## Discussion

Many previous studies have demonstrated that obese people are prone to iron deficiency, but the specific pathophysiological mechanism of this phenomenon remains incomplete. Some researchers have suggested that it is due to the intake of nutritionally unbalanced foods by obese patients. They tend to eat high-fat, high-calorie, low-nutrient foods, which can cause unbalanced intake of nutrients, including insufficient uptake of iron, thus aggravating the problem of iron deficiency. In addition, obese patients have thicker subcutaneous fat, which expands the capillary beds and leads to increased blood volume, which can also result in iron deficiency [33]. Systemic chronic inflammation is a factor of interest to a wide range of researchers, and it can decrease iron absorption by increasing the levels of hepcidin [34]. Iron homeostasis was improved in obese patients undergoing bariatric surgery, and they had significantly lower concentrations of C-reactive protein (CRP) and iron-regulated hormone in comparison to previous concentrations, presumably due to a reduction in the inflammation that may have been hindering iron absorption [35].

The duodenum is one of the major sites of iron absorption. DMT1 expressed in the duodenum is one of the key transporter proteins involved in iron uptake and the expression of duodenal DMT1 is reduced when the concentration of hepcidin is too high. At the same time, iron absorption at the tip of the intestine is inhibited, but the expression level of FPN is not altered [36, 37]. We found lower protein and mRNA expression levels of duodenal DMT1 in obese mice, but no statistically significant difference in mRNA expression of duodenal FPN between the two groups. This alteration may be due to chronic inflammation caused by obesity that increases hepcidin levels, which leads to decreased expression of duodenal DMT1, while FPN expression is not affected. Four AAs, Asp, Gln, Glu, and Gly, can increase iron absorption by increasing DMT1 expression [38], suggesting that in addition to bariatric surgery, we may also be able to prevent ORID through pharmacological interventions. In addition, we observed that HFD can also lead to morphological damage in the duodenal villi, resulting in an increased subepithelial space at the tips of some villi. This may also serve as one of the contributing factors affecting iron absorption.

Obesity, inflammation, and iron deficiency are closely related [34]. Inflammation and iron deficiency are important causes of impaired reproductive function in obese men [39–41]. Inflammation reduces the number of Leydig cells and spermatozoa, decreases sperm viability, and affects the synthesis of testicular steroid hormones [42, 43]. In addition to altering the microbiota, iron deficiency interferes with the expression of genes involved in spermatogenic activity, resulting in the impairment of male reproductive function [44]. Iron deficiency also increases oxidative stress in the testes, and iron supplementation can mitigate oxidative damage and repair testicular dysfunction [45].

MiR-135b expression has previously been found to be higher in the sperm of obese subjects [46], and it is strongly associated with both inflammation and embryonic development. Previously, researchers found that sperm from obese patients had higher numbers of miRNAs associated with both inflammation and iron metabolism. We found higher miR-135b expression in the spermatozoa of obese mice and performed bioinformatics analyses. The results of GO analysis showed that miR-135b affected reproductive function in obese male mice.

Cilia are organelles present on the cell surface, and the movement of epithelial cell cilia is involved in the movement of extracellular fluids, such as the transport of mucus in the respiratory tract. In males, motile cilia ensure that sperm can be smoothly transported from the testis to the epididymis for further functional maturation [47]. Fatty acid metabolism plays a very important role in sperm energy production, and fatty acid β-oxidation is an important source of energy during sperm maturation. Solute carrier family 22 member 14 (*SLC22A14*) regulates long-chain fatty acid β-oxidation and thus maintains energy homeostasis within spermatozoa; mutations in this gene can lead to male infertility disorders [48]. Protein phosphorylation and dephosphorylation are among the primary molecular mechanisms of sperm signaling and regulatory expression of enzymes and are also linked to sperm and egg cell signal recognition and completion of fertilization [49]. In addition, sperm capacitation cannot be achieved without tyrosine phosphorylation [50]. Sperm-egg cell fusion is one of the main processes of fertilization, and the movement of the male pronucleus to the female pronucleus of the oocyte, a process known as inward movement, is dependent on actin nucleation, which is one of the mechanisms of pronucleus migration of fertilized mouse eggs [51]. It has been shown that β-alanine, a taurine transport inhibitor, decreased reproductive hormone levels, significantly reduced sperm motility, and increased the incidence of abnormal sperm [52]. Phosphatidic acid (PA), a common phospholipid and a component of cell membranes, plays a very important role in the later stages of spermatogenesis, and phosphatidic acid levels have been associated with male infertility [53]. Glutamine and L-arginine have been proven to reduce sperm motility [54–56]. Thus, miR135b mainly affects sperm function in obese mice, including but not limited to sperm motility, sperm capacitation, sperm count, and fertilization.

We explained the cause of iron deficiency due to dietary obesity and revealed the role of iron deficiency and inflammation in the regulation of reproductive disorders.

## Conclusion

We found lower protein and mRNA expression levels of duodenal DMT1 in obese mice, and higher miR-135b expression in the spermatozoa of obese mice. In conclusion, we demonstrated that the dyfunction of DMT1 and miR-135b in the gut-testis axis induced by HFD might be an important target impairing male fertility.

## Data Availability

All data generated or analyzed during this study are included in this published article.
